# Human cytomegalovirus tegument protein UL23 promotes gastric cancer immune evasion by facilitating PD-L1 transcription

**DOI:** 10.1186/s10020-025-01114-8

**Published:** 2025-02-11

**Authors:** Shiyu Feng, Yitian Shen, Haoke Zhang, Wanfeng Liu, Weixu Feng, Xiuting Chen, Liang Zhang, Jiangli Chen, Mingdong Lu, Xiangyang Xue, Xian Shen

**Affiliations:** 1 Wenzhou Collaborative Innovation Center of Gastrointestinal Cancer in Basic Research and Precision Medicine, Wenzhou Key Laboratory of Cancer-related Pathogens and Immunity, Department of Microbiology and Immunology, School of Basic Medical Sciences, Wenzhou Medical University, Wenzhou, China; 2 Zhejiang Key Laboratory of Intelligent Cancer Biomarker Discovery and Translation, The First Affiliated Hospital of Wenzhou Medical University, Wenzhou, Zhejiang Province China; 3 The Second Affiliated Hospital, Yuying Children’s Hospital of Wenzhou Medical University, Wenzhou, China; 4 Traditional Chinese Medical Hospital of Zhuji, Zhuji, Zhejiang China; 5 Department of General Surgery, The First Affiliated Hospital of Wenzhou Medical University, Wenzhou, Zhejiang Province China

**Keywords:** HCMV, Gastric cancer, CD8^+^ T-cell, PD-1/PD-L1, UL23, PI3K-Akt pathway

## Abstract

Immune checkpoint therapy targeting PD-1/PD-L1 has shown promise in treating tumors, however, its clinical benefits are limited to a subset of gastric cancer (GC) patients. Recent research has highlighted a the correlation between PD-L1 expression and the clinical efficacy of anti-PD-1/PD-L1 therapies. Human cytomegalovirus (HCMV) has been implicated in GC, but its specific role in modulating this disease remains elusive. In this study, we analyzed clinical tissue samples using bioinformatics and real-time quantitative polymerase chain reaction (RT-qPCR). We found that GC tissues infected with HCMV presented higher PD-L1 expression compared to those without virus. Furthermore, we demonstrated that HCMV infection enhances PD-L1 expression in GC cells. Cytotoxicity assays revealed that HCMV modulates cancer immune responses via the PD-1/PD-L1 pathway. Mechanistically, we showed that HCMV activates the PI3K-Akt signaling cascade and modulates PD-L1 expression through its tegument protein UL23. Functionally, increased UL23 expression leads to elevated PD-L1 levels, which diminishes tumor cell sensitivity to T-cell-mediated cytotoxicity and triggers T-cell apoptosis. Additionally, in vivo experiments revealed that UL23-induced PD-L1 upregulation inhibits CD8^+^ T-cell infiltration and reduces the expression of inflammatory factors in tumor microenvironment, ultimately weakening antitumor immunity. Our findings reveal a novel mechanism whereby HCMV and its tegument protein UL23 contribute to cancer immunosuppression through the regulation of PD-L1 expression. This discovery may serve as a potential therapeutic target for enhancing the efficacy of cancer immunotherapy.

## Introduction

Gastric cancer (GC), the fifth most prevalent cancer worldwide and the third leading cause of cancer-related death globally, is often associated with subtle clinical symptoms, resulting in late diagnosis and poor prognosis ((Smyth et al. 2020). Immune checkpoint therapies, particularly those targeting programmed cell death protein-1 (PD-1) and its ligand PD-L1, have emerged as key strategies in cancer immunotherapy ((Topalian et al. 2020). These therapies aim to overcome tumor-induced immune suppression. PD-1/PD-L1 inhibitors have proven effective in treating various stages of GC, prolonging survival and improving quality of life (Tran et al. 2017; Yu et al. 2024). However, their efficacy remain variable, with clinical outcomes differing among individuals.

Studies have shown that PD-L1 expression is related to the success of PD-1/PD-L1 therapy ((Barber et al. 2006). Typically, active T cells express PD-1 on their surface. Upon binding to PD-L1, PD-1 transduces inhibitory signals that dampen T-cell receptor signaling, reduce T-cell proliferation, and suppress cytokine production, thus limiting tissue damage and maintaining immune tolerance (Sugiura et al. 2021; Shimizu et al. 2020). Tumors exploit this mechanism by overexpressing PD-L1, which binds PD-1 and suppresses T-cell activity, thereby evading immune surveillance (Sun et al. 2018; Ahmadzadeh et al. 2009). PD-L1 is frequently overexpressed in cancers such as lung, bladder, breast, and stomach cancers, and its high levels correlate with poor prognosis (Parry et al. 2005; Thompson et al. 2004; Ohaegbulam et al. 2015). These findings suggest that PD-L1 is a promising target for cancer treatment. Multiple factors, such as signaling pathway activation and alterations in the tumor microenvironment, regulate PD-L1 expression. Recently, viruses, especially Epstein–Barr virus (EBV) ((Wang et al. 2022), human papillomavirus (HPV) ((Ling et al. 2022), and hepatitis B virus (HBV) ((Jia et al. 2024)have been shown to upregulate PD-L1 expression, suppress T-cell activity, and promote immune tolerance, facilitating tumor immune escape.

Human cytomegalovirus (HCMV), a beta herpesvirus that primarily infects humans and other mammals, has been increasingly detected in GC tissues ((Zhang et al. 2017; Moral-Hernandez et al. 2019). Studies have reported high infection rates of HCMV in GC, with detection rates of up to 53.1% by PCR and 27.8% by next-generation sequencing (Moral-Hernandez et al. 2019; Chen et al. 2021). Our laboratory reported approximately 30% more HCMV infection in GC tissues than in adjacent areas, indicating that HCMV is a major player in GC (Zhang et al. 2017; Liu et al. 2020). HCMV is known to disrupt DNA synthesis, destabilize the host genome, and impair DNA repair mechanisms post-infection (Qian et al. 2010; Xiaofei and Kowalik 2014). Additionally, HCMV affects cell proliferation, apoptosis, differentiation, and migration, potentially converting normal cells into malignant ones (Herbein 2018, 2022). However, the role of HCMV in regulating PD-L1 expression and modulating the GC microenvironment remains largely unexplored.

In this study, we investigated the impact of HCMV infection on PD-L1 expression in GC and elucidated the underlying regulatory mechanisms. Our findings reveal a novel pathway through which HCMV contributes to immune suppression in GC, suggesting that targeting HCMV infection could enhance immunotherapy strategies for GC patients.

## Materials and methods

**Cell and virus** The human GC cell lines AGS, HGC-27 and BGC-823 were purchased from the National Cell Resource Center. All the cells were identified via short tandem repeats (STRs). The cells were cultured in RPMI 1640 (Gibco, California, USA) supplemented with 10% fetal bovine serum (FBS) (Gibco) and cultured in an incubator at 37 °C with 5% CO_2_. Human foreskin fibroblast (HFF) and mouse forestomach carcinoma (MFC) cells were cultured in Dulbecco’s modified Eagle’s medium (Gibco) supplemented with 10% FBS (Gibco). A plaque-purified derivative of the AD169 strain and Merlin strain of HCMV, which was originally obtained from the American Type Culture Collection, was used as the wild-type virus in these studies. Han-BAC-2311, the first Chinese HCMV clinical strain, Han, was a gift from Professor Luo Minhua (Wuhan Institute of Virology, China); Virus particles were partially purified from the cell culture medium by centrifugation through a sorbitol cushion, resuspended in PBS, and stored as virus stocks at -80 °C. Viral titers were determined by plaque assay on HFF cells.

**Analyses of PD-L1 expression in HCMV-negative and HCMV-positive GC tissues** Genomic data from gastric cancer (GC) patient cohorts (*N* = 45) were retrieved from the PRJEB25780 dataset in the European Nucleotide Archive (https://www.ebi.ac.uk/ena/browser/home). RNA read counts were used to analyze HCMV genomes, with a threshold of ≥ 2 reads set to distinguish HCMV-positive from HCMV-negative patients. A comparative analysis was then performed to evaluate PD-L1 expression in HCMV-negative (*N* = 33) and HCMV-positive (*N* = 12) GC groups.

**In vitro killing assay** Peripheral blood lymphocytes (PBLs) were isolated from whole blood samples of healthy men via Ficoll solution (TBD Science, Tianjin, China). CD8^+^ T cells were subsequently purified via magnetic beads (Thermo Fisher, California, USA). These lymphocytes were activated through anti-CD3/CD28 beads (Thermo Fisher) for 24 h at 37 °C and 5% CO_2_. Once activated, the lymphocytes were added to AGS cells infected with HCMV or expressing UL23 for coincubation at an effector-to-target ratio of 12:1. After 24 h, the T cells were collected and subsequently assessed for their activity and apoptosis. Moreover, the tumor cells were digested and counted via a high-content analyzer (Thermo Fisher).

**Plasmid and siRNA** The HCMV envelope genes, including UL23, UL25, UL26, UL38, UL47, UL50, UL83, UL86, UL93, UL103, and US27, were individually cloned and inserted into the pCDNA3.1-HA vector. The authenticity of all the constructs was verified through DNA sequencing. Si-UL23#1 (GCATGCGGAAGCTAAATAA) and Si-UL23#1 (GCATCACTGAATTTCTCAA) were purchased from RIBOBIO (Guangzhou, China).

**RNA extraction for RNA-seq and real-time quantitative PCR (RT-qPCR)** Total RNA was extracted with TRIzol reagent (Thermo Fisher). An RNA-sequencing (RNA-seq) analysis was conducted at Novogene Co., Ltd. (Beijing, China) on both HCMV-infected and uninfected cells at 24, 48, and 72 h post-infection to evaluate the impact of HCMV infection on the HCMV mRNA levels in GC cells. RNA sequencing was performed using the Novaseq6000 PE150 (Illumina) sequencing platform. The raw image data from the sequencing results were processed with base calling using Bcl2fastq (v2.20.0.422), followed by preliminary quality assessment (all samples had an RNA quality score above 8.7). Genes with zero expression were excluded, and the resulting data (Pass Filter Data) were further analyzed. Differentially expressed genes (DEGs) between the two groups were identified via the edgeR package, with statistical significance defined by|log2(fold change)| > 1 and a *P*-value < 0.05. For RT-qPCR, total cellular RNA was extracted via TRIzol, and subsequently, the mRNA was reverse transcribed into cDNA via the HiScript III 1st Strand cDNA Synthesis Kit (Vazyme, Nanjing, China). RT-qPCR was then performed with SYBR Premix Ex Taq II (YESEN, Shanghai, China) according to the manufacturer’s protocol. The sequences of primers used are provided in Table 1. β-actin served as the internal control for normalization purposes.

**Table 1:** Primer sequences for qRT-PCR

Primers(Human)		
CD274-Forward	5’-CTGCACTTTTAGGAGATTAGATC-3’	
CD274-Reverse	5’-CTACACCAAGGCATAATAAGATG-3’	
IL-2-Forward	5’-TGTCACAAACAGTGCACCTACT-3’	
IL-2-Reverse	5’-TCAGTTCTGTGGCCTTCTTGG-3’	
IFN-γ-Forward	5’-TCGGTAACTGACTTGAATGTCCA-3’	
IFN-γ-Reverse	5’-TCGCTTCCCTGTTTTAGCTGC-3’	
TNF-α-Forward	5’-CTGGGCAGGTCTACTTTGGG-3’	
TNF-α-Reverse	5’-CTGGAGGCCCCAGTTTGAAT-3’	
Granzyme B -Forward	5’-CCCTGGGAAAACACTCACACA-3’	
GranzymeB -Reverse	5’-GCACAACTCAATGGTACTGTCG-3’	
Perforin-Forward	5’-CAGACAGATGGAAAAGGGAGAT-3’	
Perforin-Reverse	5’-AGAATGGCGGAGGGCTTAG-3’	
UL23-Forward	5’-GTGATTACAGCGTCATTCG-3’	
UL23-Reverse	5’-GATTGTTGGCAGCAAAGAC-3’	
β-Actin-Forward	5’-CTCTTCCAGCCTTCCTTCCT-3’	
β-Actin-Reverse	5’-AGCACTGTGTTGGCGTACAG-3’	
Primers(Mouse)		
IFN-γ-Forward	5’-ACTCAAGTGGCATAGATGTGGAAGA-3’	
IFN-γ-Reverse	5’-ATGACGCTTATGTTGTTGCTGATGG-3’	
GzmB-Forward	5’-AGAACAGGAGAAGACCCAGCAAGT-3’	
GzmeB-Reverse	5’-CCAACCAGCCACATAGCACACAT-3’	
CCL9-Forward	5’-TCATTGCTACACTGAAGAACGGAGA-3’	
CCL9-Reverse	5’-TCCTTGAACGACGACGACTTTGG-3’	
CXCL10-Forward	5’-CGCTGCAACTGCATCCATATCG-3’	
CXCL10-Reverse	5’-CGGATTCAGACATCTCTGCTCATCA-3’	
β-Actin-Forward	5’-ACTATTGGCAACGAGCGGTTCC-3’	
β-Actin-Reverse	5’-GGCATAGAGGTCTTTACGGATGTCA-3’	

(located in Materials and methods)

**Western blot (WB) assay** The cells were lysed with RIPA buffer (Beyotime, Shanghai, China) containing a proteinase inhibitor cocktail (MCE, New Jersey, USA) and a phosphatase inhibitor cocktail (Beyotime). The protein concentrations of the samples were estimated via a BCA assay kit (Beyotime). Protein samples were separated via 10% SDS-PAGE, and the separated proteins were subsequently transferred to a PVDF membrane (Bio-Rad, Calif, USA). The membrane was blocked with 5% milk (Beyotime, Shanghai, China) for 1 h at room temperature and probed with primary antibodies (anti-PD-L1, 1:1000; anti-HA, 1:1000; anti-PI3K, 1:1000; Cell Signaling Technology, MA, USA; anti-p-PI3K, 1:1000; Abcam, Cambridge, Britain; anti-p-AKT, 1:1000; anti-AKT, 1:1000; HUABIO, Hangzhou, China) at 4 °C overnight. Next, the membranes were incubated with a peroxidase-conjugated secondary antibody for 1 h at room temperature. GAPDH (1:10000, HUABIO) was used as the protein loading control. The blots were developed via an eECL Western Blot Kit (Thermo Fisher) according to the manufacturer’s instructions. The intensities of the WB bands were quantified via ImageJ software.

**Surface flow cytometry analysis of PD-L1.** The cells were collected, washed with PBS twice and resuspended in 100 µL of PBS containing primary antibodies (anti-human CD274, Invitrogen, Calif, USA; anti-mouse PD-L1, BioLegend, Calif, USA). The reaction was performed at room temperature for 15 min and terminated by the addition of 400 mL of PBS. The cells were then analyzed via flow cytometry via a BD FACS Canto II instrument. The data were analyzed via FlowJo_V10 software. To prepare the tumor cell suspension, the tumor tissue was cut into small pieces, ground, and digested with type IV collagenase and DNase I (Roche, Basel, Switzerland) at 37 °C for 30 min. The suspensions were filtered through 70 μm nylon cell strainers. After centrifugation (2000 rpm, 5 min) and resuspension in PBS, the remaining steps were similar to those of the cells.

**Immunohistochemistry (IHC).** The paraffin-embedded slides were deparaffinized, rehydrated and then subjected to antigen retrieval by treatment with sodium citrate in a microwave oven for 2 min. The slides were further incubated in a 3% H_2_O_2_ solution to block endogenous peroxidase activity. After being blocked with 5% goat serum, the tissues were incubated with primary antibodies against CD8 (1:200, BioLegend) at 4 °C overnight. After rinsing with PBS, the slides were incubated with a biotin-conjugated secondary antibody (1:50, Invitrogen) at room temperature for 30 min, washed, and incubated with HRP-conjugated streptavidin. Hematoxylin was used to counterstain the slides. The cells were observed under an upright fluorescence microscope (BX 53; Olympus, Japan).

**Animal studies.** A total of 2 × 10^6^ UL23-overexpressing MFC cells (UL23-MFCs) or control MFC cells (Ctrl-MFCs) in 100 µL of buffered saline were subcutaneously injected into 5–7-week-old 615 mice (*n* = 6 per group; Vital River, Beijing, China). Moreover, 2 × 10^6^ UL23-overexpressing BGC-823 cells (UL23-BGC-823) or control BGC-823 cells (Ctrl-BGC-823) in 100 µL of buffered saline were subcutaneously injected into BALB/c nude mice (Vital River). Tumor size was measured every 2 d by two independent observers via calipers fitted with a Vernier scale. The tumor volume was calculated on the basis of three perpendicular measurements. Once the mice were sacrificed, the tumors were photographed and further fixed for immunohistochemical staining and RT-qPCR, and the tumors were dissociated into single cells for flow cytometry.

**Statistical analysis**. Statistical analysis was performed using Prism software (GraphPad 9.0.0, San Diego, CA, USA). Unpaired Student’s t-test was applied to compare mean differences between two groups, while one-way ANOVA with multiple comparisons was used for comparisons among multiple groups. Data are presented as Means ± SEM (standard error of the mean). A p-value of < 0.05 was considered statistically significant. All experiments were conducted independently in triplicate.

## Results

### HCMV infection increased PD-L1 expression in GC tissues and cells

**Fig. 1:**
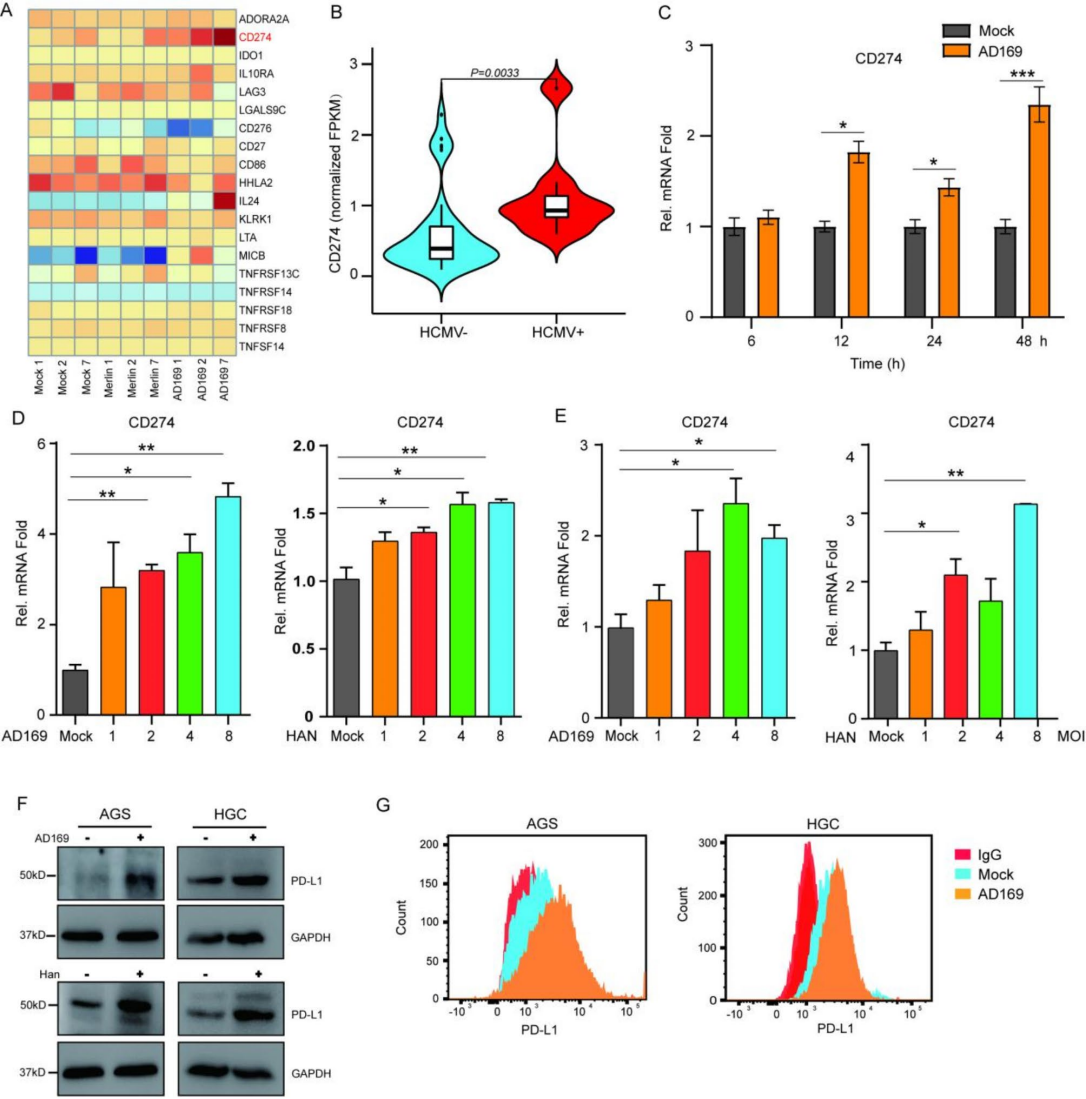
HCMV infection increased PD-L1 expression in GC cells (**A**) A heatmap showing two HCMV strains, AD169 and merlin, and the effects of various cell membrane molecules after different durations of infection in GC cells, with the depth of color indicating high or low correlation. “Mock 1,” “Mock 2,” and “Mock 7” represent AGS cells uninfected with HCMV for 1, 2, or 7 days, respectively. “Merlin 1,” “Merlin 2,” and “Merlin 7” represent AGS cells infected with the Merlin strain for 1, 2, or 7 days, respectively. “AD169 1,” “AD169 2,” and “AD169 7” represent AGS cells infected with the AD169 strain for 1, 2, or 7 days, respectively. (**B**) Box plots illustrating the expression level of PD-L1 in HCMV-positive and HCMV-negative GC tissues. (**C**) RT-qPCR analysis was conducted on AGS cells that were either uninfected or infected with AD169 (MOI = 1) for specified time intervals. (**D**) AGS or (**E**) HGC-27 cells were infected with AD169 or Han-BAC at MOIs of 1, 2, 4, and 8 for 48 h before RT-qPCR analysis. (**F**) WB analysis was performed on AGS and HGC-27 cells that were either uninfected or infected with AD169 or Han-BAC strains (MOI = 1) for 48 h. (**G**) Flow cytometry was used to quantify surface PD-L1 levels in AGS and HGC-27 cells following 48 h of infection with AD169. Statistical significance is denoted as follows: * *P* < 0.05, ** *P* < 0.01

Tumor cells evade the surveillance and attack of the immune system through specific membrane proteins on their cell surfaces. To explore whether HCMV affects the immune evasion characteristics of tumors, we examined the impact of HCMV on several membrane proteins through RNA-Seq. As shown in the heatmap (Fig. 1A), infection with two distinct HCMV strains, Merlin and AD169, increased PD-L1 (encoded by CD274) expression in a time-dependent manner, with no significant changes in other membrane proteins or receptors. We subsequently analyzed 45 GC tissues and divided them into HCMV-positive and HCMV-negative groups, and the results revealed that PD-L1 expression levels in HCMV-positive gastric cancer tissues (Mean ± SEM: 0.893 ± 0.186, *N* = 12) were significantly higher compared to HCMV-negative tissues (Mean ± SEM: 0.621 ± 0.106 *N* = 33). The difference in PD-L1 expression between HCMV-positive and HCMV-negative tissues was statistically significant (*P* = 0.0033) (Fig. 1B). To further elucidate the effect of HCMV on PD-L1 expression, AGS cells were infected or not infected with AD169 at various time points. AD169 upregulated PD-L1 expression in a time-dependent manner, with the most prominent regulation occurring 48 h post-infection (Fig. 1C). Additionally, infection of AGS and HGC-27 cells with the AD169 and Han-BAC strains, respectively, led to a significant increase in PD-L1 expression as the multiplicity of infection (MOI) increased in both cell lines (Fig. 1D-E). Further analysis via WB and flow cytometry revealed substantial increases in PD-L1 protein levels in both the total protein extracts and the membrane fractions of HCMV-infected GC cells (Fig. 1F-G). These findings underscore the influence of HCMV on PD-L1 expression in GC cells.

### HCMV infection regulates T-cell activation and apoptosis by promoting PD-L1 expression in GC

**Fig. 2:**
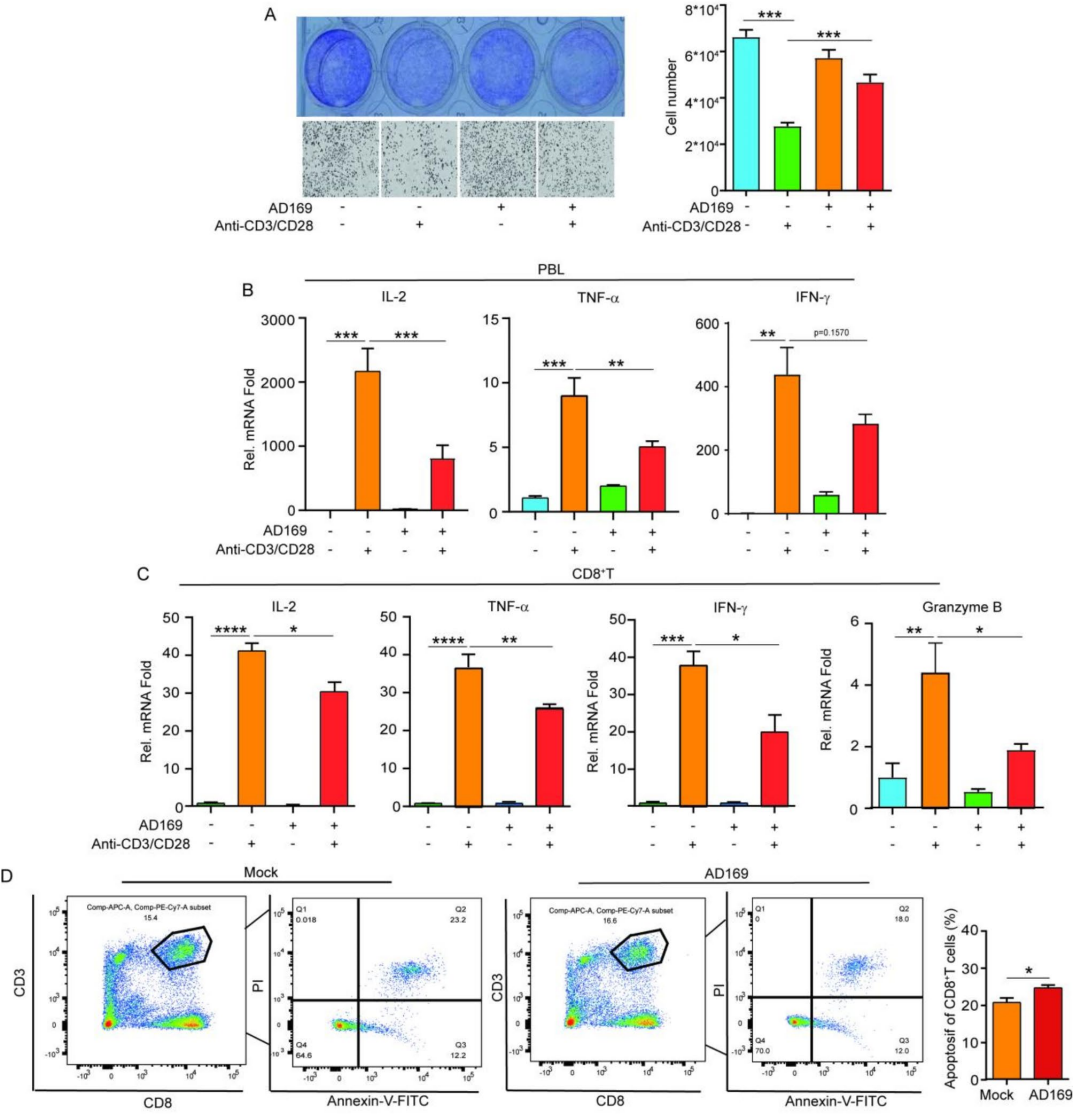
HCMV infection suppresses T-cell activity and induces apoptosis (**A**) PBLs activated with anti-CD3/CD28 exhibited reduced efficacy in eliminating AGS cells infected with AD169, with an effector-to-target cell ratio of 12:1 for 24 h. The upper panel shows Coomassie Brilliant Blue staining of the cells; the lower panel shows a microscopy image; and the right-hand panel presents the statistical analysis of the cell counts. (**B**) RT-qPCR analysis of IL-2, IFN-γ, and TNF-a expression in anti-CD3/CD28-stimulated PBLs, comparing AD169-infected versus uninfected AGS cell for 24 h. (**C**) RT-qPCR analysis of IL-2, TNF-a, IFN-γ, and GzmB expression in anti-CD3/CD28-stimulated CD8^+^ T cells cocultured with AD169-infected or uninfected AGS cells for 24 h. (**D**) Apoptosis levels of CD8^+^ T cells were assessed via flow cytometry. CD8^+^ T cells were cocultured with AGS cells infected with or without HCMV at a 12:1 ratio for 24 h. The combined percentage of Annexin V-positive and PI-positive cells was considered the proportion of apoptotic CD8^+^ T cells. The right-hand panel shows the quantitative plot of the proportion of apoptotic CD8^+^ T cells. Statistical significance is indicated as follows: * *P* < 0.05, ** *P* < 0.01, *** *P* < 0.001

To investigate whether HCMV infection affects T-cell-mediated killing of GC cells, activated PBLs were cocultured with AD169-infected or uninfected GC cells. The results showed that activation of PBLs with CD3/CD28 stimulation reduced T-cell killing of GC cells upon HCMV infection (Mean ± SEM: 46,676 ± 3,403 in the AD169 infected group vs. 27,786 ± 1,552 in the uninfected group, *P* < 0.001) (Fig. 2A). HCMV infection significantly suppressed PBL activation, as evidenced by a marked decrease in IL-2 production (Mean ± SEM: 820.9 ± 223.9 in AD169-infected cells vs. 2,169 ± 353.5 in uninfected cells, *P* < 0.001), TNF-α expression (Mean ± SEM: 5.049 ± 0.4346 in AD169-infected cells vs. 8.998 ± 1.379 in uninfected cells, *P* < 0.01), and a trend toward reduced IFN-γ production (Mean ± SEM: 285.3 ± 31.39 in AD169-infected cells vs. 440.3 ± 83.45 in uninfected cells, *P* = 0.1570) (Fig. 2B). These results show that HCMV infection of GC cells inhibits the secretion of key cytokines during T-cell activation. Similarly, coincubation with HCMV-infected GC cells also reduced the expression levels of IL-2 (Mean ± SEM: 31.04 ± 4.127 in AD169 infected cells vs. 41.18 ± 2.009 in uninfected cells, *P* < 0.05), TNF-a (Mean ± SEM: 27.32 ± 1.617 in AD169 infected cells vs. 36.50 ± 3.365 in uninfected cells, *P* < 0.01), IFN-γ (Mean ± SEM: 21.24 ± 5.755 in AD169 infected cells vs. 37.80 ± 3.365 in uninfected cells, *P* < 0.05) and Granzyme B (GzmB) (Mean ± SEM: 1.891 ± 0.1995 in AD169 infected cells vs. 4.408 ± 0.9954 in uninfected cells, *P* < 0.05) in CD8^+^ T cells (Fig. 2C). We also examined apoptosis in cocultured CD8 + T cells via flow cytometry. As shown in Fig. 2D, HCMV infection increased the apoptotic ratio and increased the resistance of GC cells to CD8^+^ T-cell-mediated cytolysis. These findings confirm that HCMV infection of GC cells impairs the cytotoxicity and immune activity of CD8^+^ T cells within the GC microenvironment, promoting CD8^+^ T-cell apoptosis.

**Fig. 3:**
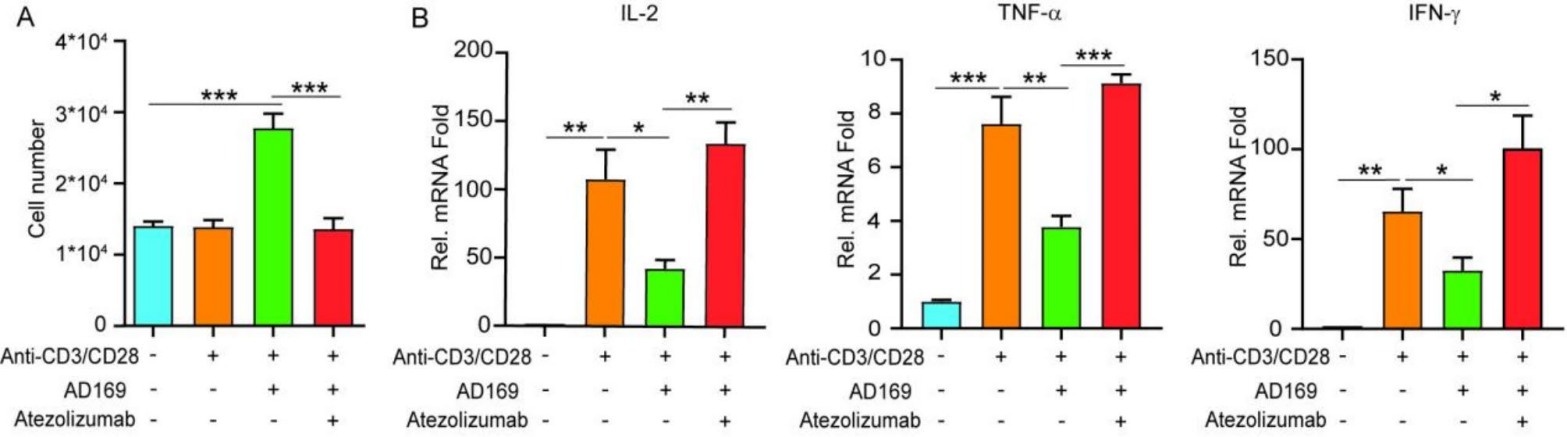
The suppression of CD8 **+** T-cell antitumor activity by HCMV relies on PD-L1 expression (**A**) The application of a PD-L1 inhibitor (atezolizumab) enhanced the cytotoxic efficacy of CD8^+^ T cells. After coincubation with anti-CD3/CD28-activated CD8^+^ T cells, the cells were treated with atezolizumab or DMSO for 24 h, and the subsequent survival rate of the AGS cells was assessed. (**B**) RT-qPCR was used to measure the expression levels of IL-2, TNF-α, and IFN-γ mRNAs in CD8^+^ T cells cocultured with AGS cells with atezolizumab or DMSO treatment. Statistical significance is denoted as follows: * *P* < 0.05, ** *P* < 0.01, *** *P* < 0.001

Furthermore, we investigated the relationship between the impact of HCMV on T-cell activity and PD-L1 expression. In the context of AD169 infection, we incorporated the PD-L1 inhibitor atezolizumab or DMSO into our coculture system. Notably, PD-L1 inhibition led to a significant reduction in tumor cell count (Mean ± SEM: 13,743 ± 1,784 in the atezolizumab group vs. 27,864 ± 2,257 in the DMSO group, *P* < 0.001) (Fig. 3A), whereas the uninfected cohort remained unaffected. Consistently, inhibiting PD-L1 expression restored crucial cytokine levels, including IL-2 (Mean ± SEM: 134.6 ± 15.50 in atezolizumab group vs. 42.83 ± 6.335 in DMSO group, *P* < 0.01), TNF-a (Mean ± SEM: 9.127 ± 0.3247 in atezolizumab group vs. 3.777 ± 0.4135 in DMSO group, *P* < 0.001) and IFN-γ (Mean ± SEM: 100.6 ± 18.10 in atezolizumab group vs. 32.53 ± 7.219 in DMSO group, *P* < 0.05) in CD8^+^ T cells (Fig. 3B). Collectively, these findings underscore the mechanism whereby HCMV impairs T-cell antitumor efficacy by upregulating PD-L1 expression in GC cells.

### The HCMV tegument protein UL23 upregulates PD-L1 expression in GC cells

**Fig. 4:**
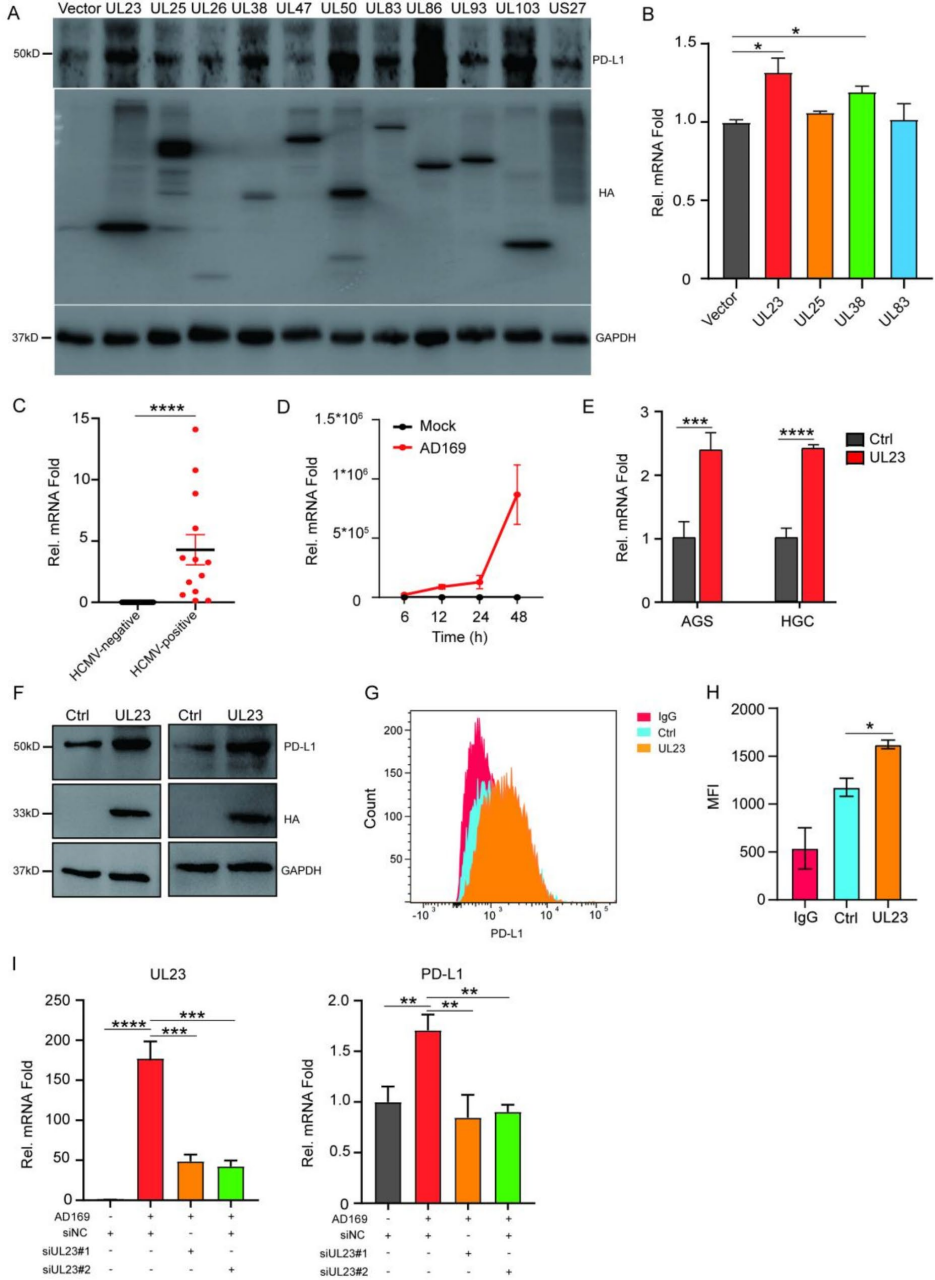
The HCMV tegument protein UL23 upregulates PD-L1 expression in GC cells (**A**) WB analysis of the effects of various tegument proteins on PD-L1 expression. (**B**) RT-qPCR analysis of PD-L1 expression following the transfection of different plasmids into AGS cells. (C) Comparison of UL23 mRNA levels in HCMV-positive versus HCMV-negative GC tissues. (**D**) Temporal analysis of UL23 mRNA expression post-HCMV infection at various time points. (**E**) After 48 h of UL23 transfection in AGS and HGC-27 cells, PD-L1 levels were quantified via RT-qPCR, and (**F**) total cell lysates were analyzed by WB. Flow cytometry was used to quantify surface PD-L1 levels in UL23 stable expression AGS cells (**G**), and the results of the quantitative analysis are provided in (**H**). (**I**) RT-qPCR analysis of UL23 (left panel) and PD-L1 expression (right panel) following transfection with two distinct UL23-targeting siRNAs in HCMV infection. Statistical significance is indicated as follows: *P < 0.05, **P < 0.01, ***P < 0.001. The data represent three independent experiments with similar results

Numerous biochemical and biological traits of viruses are intricately tied to their products and compositional makeup. As shown in Fig. 1C, the expression of PD-L1 in GC cells infected with HCMV was significantly elevated at approximately 12 h post-infection. These findings prompted us to hypothesize that the upregulation of PD-L1 in GC cells by HCMV may be a direct consequence of its composition. Notably, the viral envelope protein acts as a pivotal portal for viral entry into cells ((Plemper 2011), simultaneously eliciting immune responses within the host. To further explore this phenomenon, we constructed 11 HCMV envelope proteins that are highly expressed in GC and carefully investigated their potential role in HCMV-mediated PD-L1 expression. Among the successfully expressed plasmids, UL23 and UL38 emerged as modulators of PD-L1 expression, with UL23 exhibiting the most pronounced effect (Mean ± SEM: 1.321 ± 0.09921 in cells transfected with UL23 vector vs. 1.000 ± 0.02653 in cells transfected with empty vector, *P* = 0.00354) (Fig. 4A-B). These findings underscore the pivotal role of UL23 in regulating PD-L1 expression. Furthermore, we observed a marked increase in UL23 expression in HCMV-positive GC tissues compared with that in their negative counterparts (Fig. 4C). Additionally, when AGS cells were infected with AD169, UL23 expression gradually increased, peaking at 48 h post-infection (Fig. 4D). This temporal pattern aligns with the observed increase in PD-L1 levels in HCMV-infected GC cells, reinforcing the correlation between UL23 and PD-L1 regulation.

To validate the influence of HCMV UL23 on PD-L1 expression in GC, we transfected AGS and HGC-27 cells with the UL23 vector for 48 h, and PD-L1 expression was significantly increased at both the mRNA (AGS: 2.377 ± 0.1355 in cells transfected with UL23 vector vs. 1.000 ± 0.1251 in cells transfected with empty vector, *P* = 0.0003) (HGC: 2.403 ± 0.1162 in cells transfected with UL23 vector vs. 1.000 ± 0.08665 in cells transfected with empty vector, *P* < 0.0001) (Fig. 4E) and protein levels (Fig. 4F). Further analysis via flow cytometry revealed higher PD-L1 protein levels in the membrane fraction of UL23 vector overexpressed GC cells (MFI: 1,623 ± 32.00 in UL23 vector overexpressed GC cells vs. 1,175 ± 67.00 in control cells, *P* = 0.0264) (Fig. 4G-H). Moreover, when AGS cells were pretreated with UL23 siRNA (siUL23) prior to HCMV infection, PD-L1 expression was significantly reduced. The results showed (Mean ± SEM: 0.8455 ± 0.1305 in the siUL23#1 group vs. 1.707 ± 0.09007 in the siNC group, *P* = 0.0056; Mean ± SEM: 0.9024 ± 0.04034 in the siUL23#2 group vs. 1.707 ± 0.09007 in the siNC group, *P* = 0.0012) (Fig. 4I). These findings suggest that UL23 plays a key role in HCMV-induced PD-L1 expression.

4. **UL23 mediates the immunosuppressive function of CD8** ^**+**^ **T cells.**

**Fig. 5:**
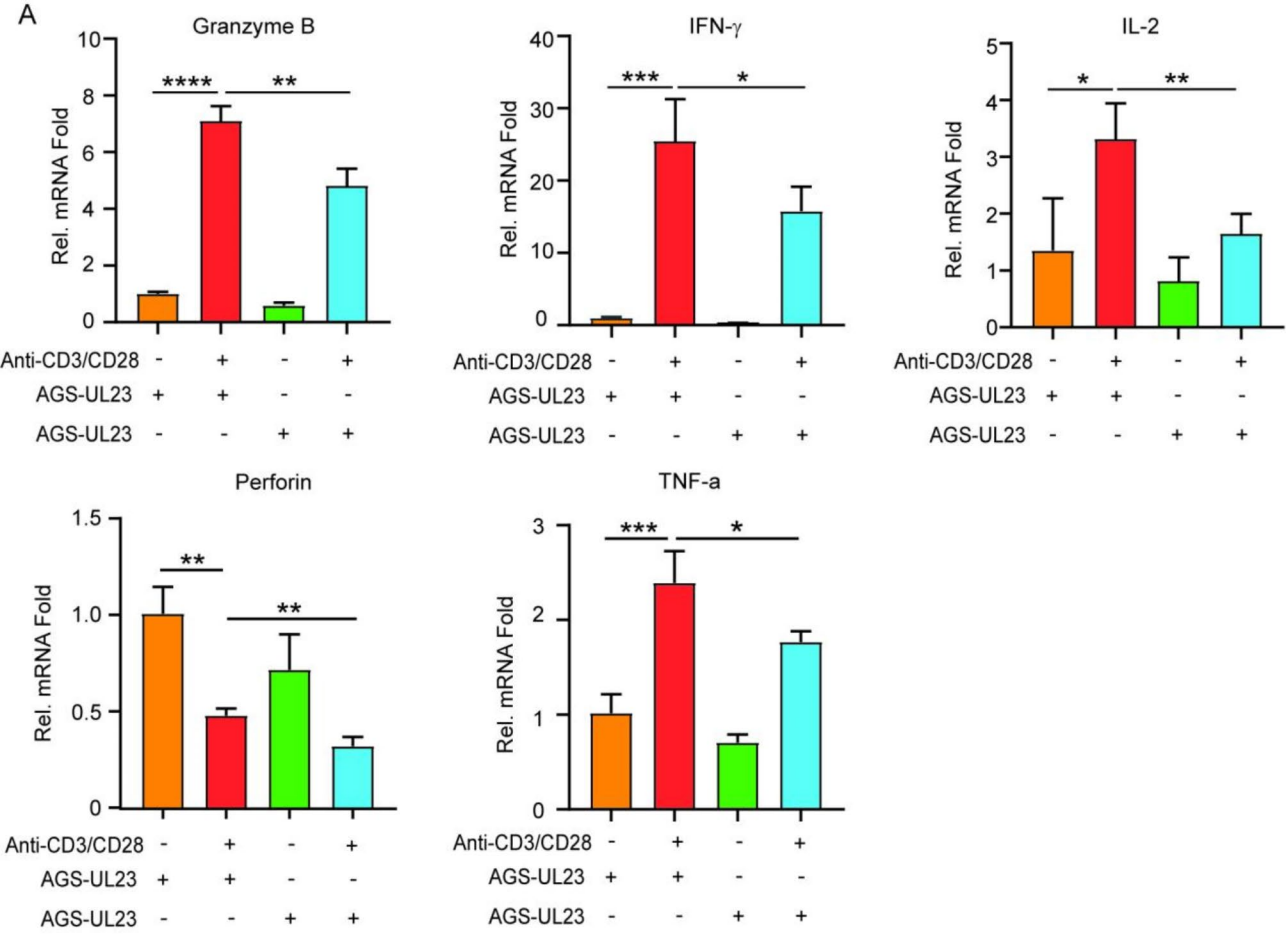
UL23 inhibits T-cell activity by increasing PD-L1 expression (**A**) RT-qPCR analysis of IL-2, TNF-α, IFN-γ, and GzmB mRNA levels in CD8^+^ T cells stimulated with anti-CD3/CD28 in the presence of either Ctrl-AGS or UL23- AGS cells. Statistical significance is indicated as follows: * *P* < 0.05, ** *P* < 0.01

To determine whether UL23 affects the functional status of tumor-infiltrating CD8^+^ T cells, we cocultured AGS cells stably overexpressing UL23 or the control vector with CD8^+^ T cells. Compared with those in the control group, HCMV UL23 expression significantly reduced CD8^+^ T-cell activation, as evidenced by decreased GzmB expression (Mean ± SEM: 4.821 ± 0.3091 in UL23-AGS cells vs. 7.100 ± 0.2720 in Ctrl-AGS cells, *p* < 0.01), IFN-γ expression (Mean ± SEM: 15.80 ± 1.673 in UL23-AGS cells vs. 25.48 ± 3.367 in Ctrl-AGS cells, *P* < 0.05), IL-2 expression (Mean ± SEM: 1.650 ± 0.1785 in UL23-AGS cells vs. 3.318 ± 0.3231 in Ctrl-AGS cells, *P* < 0.01), Perforin expression (Mean ± SEM: 0.3194 ± 0.02371 in UL23-AGS cells vs. 0.4776 ± 0.01821 in Ctrl-AGS cells, *P* < 0.01) and TNF-a expression (Mean ± SEM: 1.768 ± 0.05637 in UL23-AGS cells vs. 2.391 ± 0.1678 in Ctrl-AGS cells, *P* < 0.05) (Fig. 5A).

5. **The UL23/PI3K/Akt signaling pathway upregulates PD-L1 expression.**

**Fig. 6:**
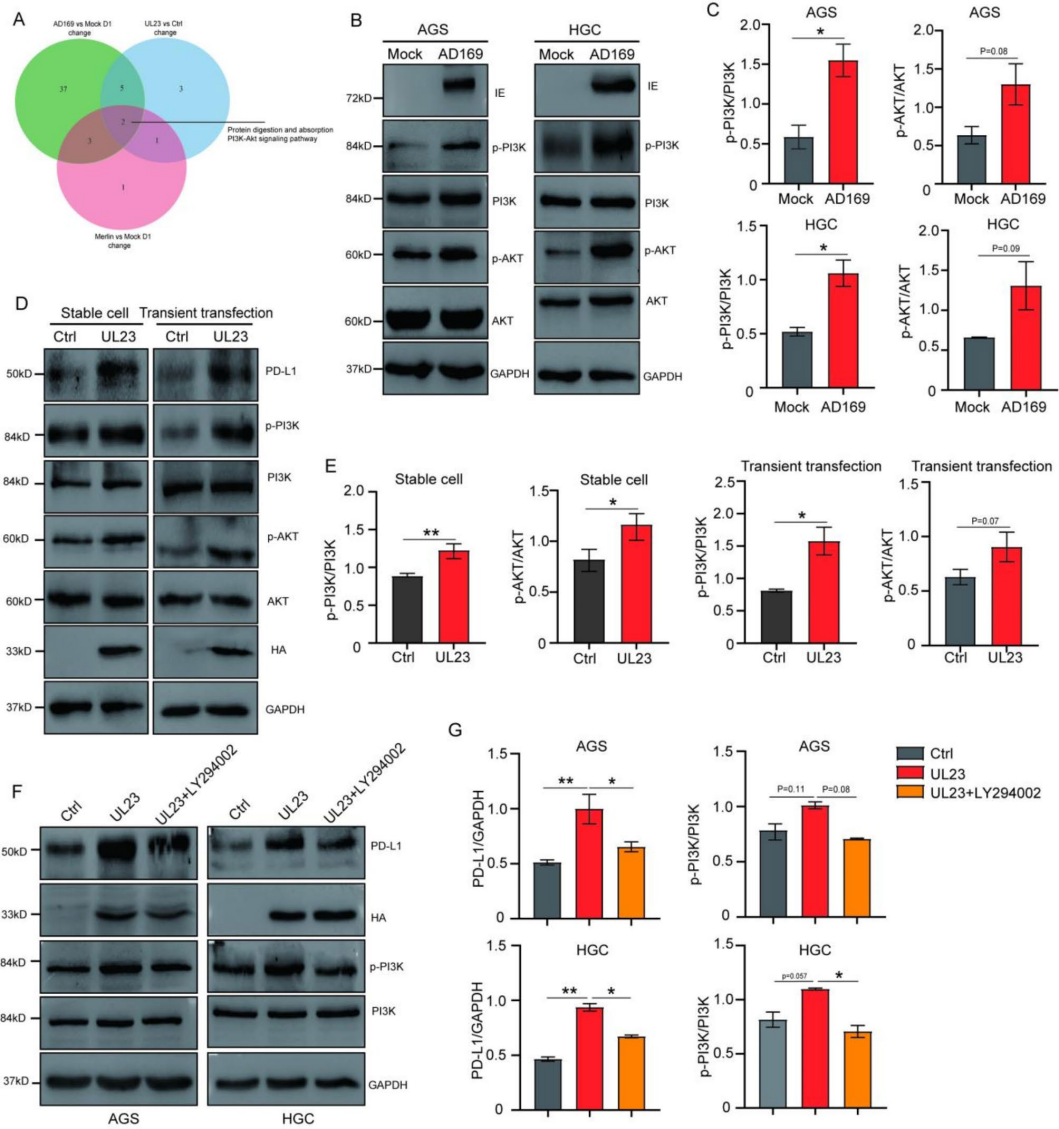
UL23 upregulates PD-L1 expression via the PI3K/Akt pathway (**A**) Venn diagram showing that HCMV (AD169 and Merlin strains) infection and UL23 expression regulate the PI3k-Akt signaling pathway. (**B**) WB analysis of PI3K-Akt signaling in AGS and HGC-27 cells infected with HCMV or not. (**C**) A quantified statistic for the results in panel B. (**D**) WB analysis of the impact of transient UL23 transfection (left panel) and stable UL23 expression (right panel) on the PI3K-Akt signaling pathway. (**E**) Quantification of the results in panel D. (**F-G**) To assess the specificity of UL23-mediated PI3K-Akt activation, LY294002, a PI3K inhibitor, was applied to AGS and HGC-27 cells transfected with UL23. Subsequent WB analysis was conducted to monitor PI3K-Akt signaling. G represents the repetition and quantification of the data in panel F. Statistical significance is denoted as follows: * *P* < 0.05, ** *P* < 0.01, *** *P* < 0.001

To analyze the differential signaling pathways between HCMV infection and non-infection, as well as UL23 expression and non-expression in AGS cells, Venn diagram analysis revealed shared signaling pathway changes between HCMV infection and UL23 overexpression, particularly those involving the PI3K-Akt pathway (Fig. 6A). The PI3K-Akt pathway can be activated by gene mutations and growth factors, and it is recognized as one of the classical oncogenic singnaling pathways that likely induces PD-L1 expression. The activation of the PI3K-Akt pathway has been well documented for its ability to induce PD-L1 expression in various cancer types, including small cell lung cancer ((Quan et al. 2022). In line with the RNA-seq results, the results from the WB analysis indicated a significant increase in the expression levels of p-PI3K/PI3K and p-Akt/Akt in both AGS and HGC-27 cells following HCMV infection (Fig. 6B-C). Similarly, the overexpression of UL23 also significantly augmented the activation of PI3K-Akt (Fig. 6D-E).

To validate the role of PI3K-Akt as the crucial pathway activated by HCMV and UL23, specific inhibitors of the PI3K kinase LY294002 were employed. The results conclusively revealed that LY294002 significantly inhibited UL23-induced PD-L1 expression (Fig. 6F-G). This compelling evidence suggests that HCMV may increase PD-L1 expression by producing UL23 and subsequently activating the PI3K-Akt signaling cascade.

6. **UL23 promoted immune escape in GC in vivo.**

**Fig. 7:**
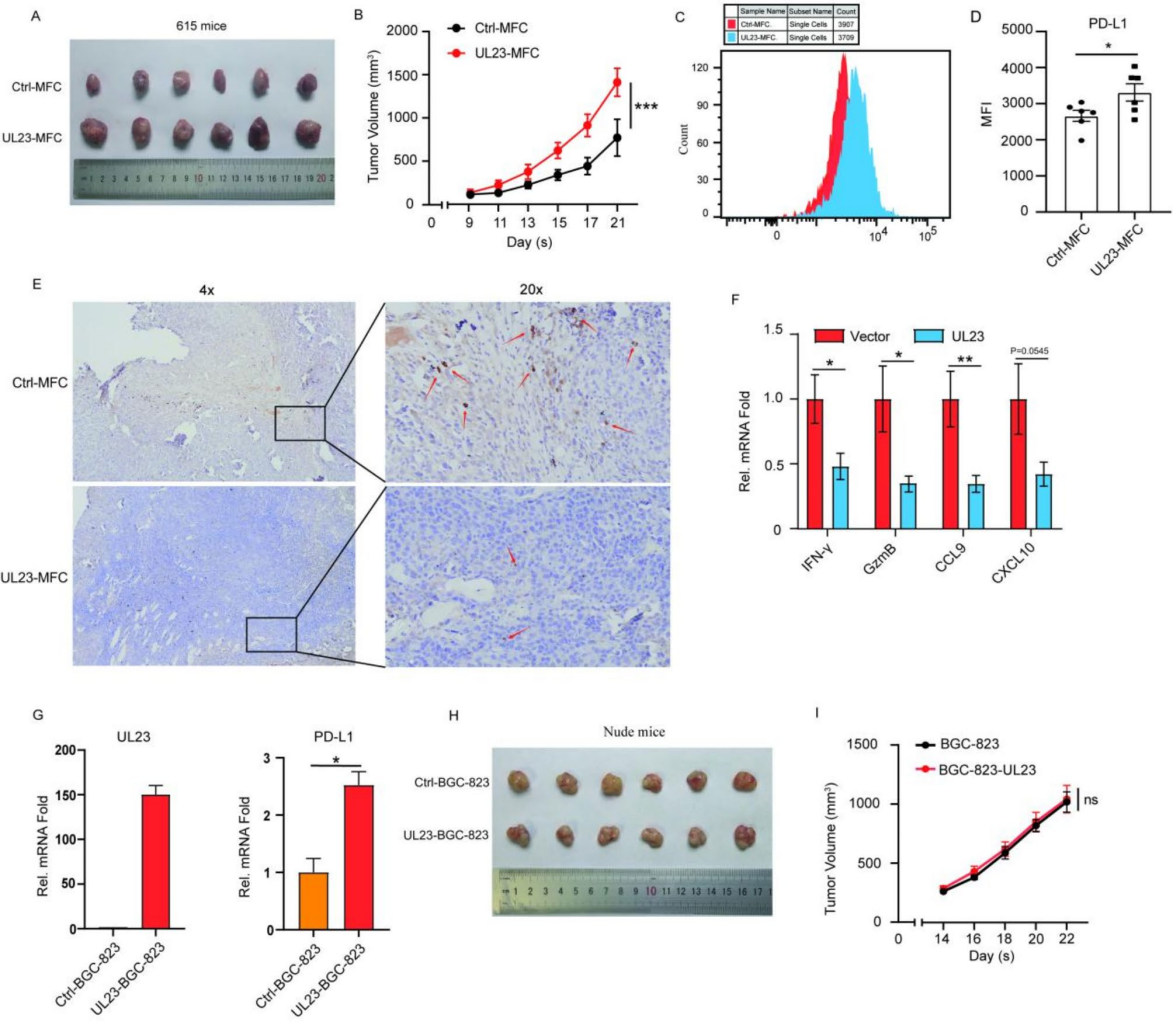
UL23 enhances GC growth and immune evasion in vivo via upregulation of PD-L1 expression Significant acceleration of gastric tumor growth was observed in 615 mice overexpressing UL23. UL23-MFCs or Ctrl-MFCs (2 × 10^6^ cells/mouse) were subcutaneously injected into 6-8-week-old female 615 mice. Tumor dimensions were recorded daily from day 7 postinoculation and are presented as the mean ± SEM (*n* = 6 mice/group). Representative tumor images (**A**) and tumor growth rates (**B**) for each group are shown. Flow cytometry was used to detect PD-L1 expression in mouse tumor cells (**C**), and the results of the quantitative analysis are provided in (**D**). (**E**) Elevated mRNA levels of IFN-γ, GzmB, CXCL9, and CXCL10 were observed in the UL23-MFC group (*n* = 5 or 6 per group). (**F**) IHC analysis of CD8^+^ T cells in the GCs of 615 mice. (**G**) UL23-BGC-823 expression was confirmed by RT-qPCR, which was used to measure UL23 and PD-L1 mRNA levels. (**H-I**) UL23-BGC-823 or Ctrl-BGC-823 cells (2 × 106 cells/mouse) were subcutaneously injected into 6–8-week-old female nude mice. Representative GC tumor images (**H**), tumor growth rates, and volumes (**I**) are presented. The data are shown as the means ± SEM. Statistical significance is indicated as follows: *P < 0.05, **P < 0.01

To investigate whether UL23 promotes immune evasion of GC in vivo, mouse UL23-MFCs were inoculated into immunocompetent mice (615 mice) to establish a xenograft model. After a seven-day observation period, palpable tumor formation was observed, and the tumor volume was documented on day nine. Notably, the UL23 group exhibited faster tumor growth, with the tumors being significantly larger in the UL23-MFC group compared to the Ctrl-MFC group at 21 days post-subcutaneous injection (Tumor volume (mm³) on day 14: 1,412 ± 161.9 in UL23-MFC vs. 770.4 ± 213.5 in Ctrl-MFC, *P* < 0.001) (Fig. 7A-B). Flow cytometry analysis revealed that tumors from the UL23-MFC group had increased PD-L1 fluorescence intensity on the surface of tumor cells (MFI: 3,313 ± 240.5 in UL23-MFC vs. 2,666 ± 154.0 in Ctrl-MFC, *P* = 0.0468) (Fig. 7C-D). Moreover, the number of infiltrating CD8^+^ T cells (Fig. 7E) and the mRNA levels of cytokines were lower in the UL23-MFC group than in the Ctrl-MFC group. These cytokines include IFN-γ (Mean ± SEM: 0.4937 ± 0.09847 in UL23-MFC group vs. 1.000 ± 0.1869 in Ctrl-MFC group, *P* = 0.0294), GzmB (Mean ± SEM: 0.3402 ± 0.06057 in UL23-MFC group vs. 1.000 ± 0.2548 in Ctrl-MFC group, *P* = 0.0202), CXCL9 (Mean ± SEM: 0.3457 ± 0.06501 in UL23-MFC group vs. 1.000 ± 0.2144 in Ctrl-MFC group, *P* = 0.0096), and CXCL10 (Mean ± SEM: 0.4203 ± 0.09229 in UL23-MFC group vs. 1.000 ± 0.2722 in Ctrl-MFC group, *P* = 0.0545) (Fig. 7F). These findings suggest that UL23 promotes tumorigenesis by enhancing the immune evasion ability of GC cells.

It is intriguing to determine whether HCMV modulates PD-L1 expression to promote GC progression in an immune-dependent manner. To explore this, we used immunocompromised nude mice, which lack mature functional T cells and adaptive immune responses, to examine the role of UL23-mediated inhibition of T cell immune function in tumor growth. We inoculated Ctrl-BGC-823 or UL23-BGC-823 cells into nude mice. Prior to tumor implantation, we confirmed at the mRNA level that UL23 promotes PD-L1 expression in BGC-823 cells (Mean ± SEM: 2.523 ± 0.2338 in UL23-BGC-823 cells vs. 1.000 ± 0.2425 in Ctrl-BGC-823 cells, *P* = 0.0107) (Fig. 7G). Tumor volume was recorded from day 14 post-implantation. In nude mice, no significant differences in tumor growth or volume were observed between the UL23-BGC-823 group and the Ctrl-BGC-823 group (Fig. 7H-I), indicating that the immune microenvironment contributes to the reduced growth of UL23-overexpressing tumors.

## Discussion

Both viruses and tumors harness PD-L1 to dampen T-cell activity and proliferation, thereby diminishing the body’s capacity to eliminate them and facilitating immune evasion, which undermines the effectiveness of immunotherapy. Research has revealed intricate interactions between HCMV infection and T cells. While HCMV infection triggers T-cell immune responses, it concurrently leads to reduced T-cell activity, depletion of cell numbers, and cellular dysfunction, thereby compromising the immune system ((Klenerman and Oxenius 2016; Zangger and Oxenius 2022). On the basis of these insights, we investigated the immunomodulatory effects of HCMV on cancer cells and confirmed that HCMV indeed upregulates PD-L1 expression in GC cells, modulates T-cell activity, and reduces T-cell infiltration into tumor cells, thereby accelerating GC progression. This finding implies that HCMV-infected GC patients may derive greater benefits from immunotherapy than their uninfected counterparts. Consequently, detecting HCMV infection status or associated immune markers could facilitate the prediction of patient responsiveness to immunotherapy. As previously suggested, HCMV may sustain latent infection by continuously inducing PD-L1 expression ((Hu et al. 2020). Thus, therapies aimed at the PD-1/PD-L1 axis have emerged as promising options for treating HCMV infections.

To date, HCMV has been associated with various malignant diseases, but direct evidence linking HCMV to the development of these cancers is lacking ((Söderberg-Nauclér 2008; Hunter-Schlichting et al. 2021; Söderberg-Nauclér 2006). Some HCMV gene products, including IE, US28, UL36-UL38, UL97, and gB, have been shown to inhibit tumor cell apoptosis, alter the cell cycle, and promote tumor cell infiltration (Cobbs et al. 2008, 2014; Soroceanu et al. 2011; Colberg-Poley 1996; Iwahori et al. 2017). In our study, we revealed that the HCMV membrane protein UL23 mediates the immune escape of GC cells. UL23, a component of the viral envelope, is expressed in the cytoplasm of HCMV-infected cells. Research on UL23 is limited, and current studies primarily focus on its role in modulating the interferon response during HCMV infection. By disrupting STAT1 phosphorylation, inhibiting the Nmi-STAT1 interaction, and suppressing interferon gene activation, UL23 effectively dampens the type I interferon (IFN-I) response (Wang et al. 2023; Feng et al. 2018, 2021). These findings underscore the distinctive potential of UL23 in regulating interferon responses. Here, we explored the function of UL23 in tumor immunity. Interestingly, despite its capacity to increase PD-L1 expression in fibroblasts by activating the type II interferon response (IFN-II) ((Yuan et al. 2024), we observed no activation of IFN-I or IFN-II responses in GC cells overexpressing UL23 (data not shown). These findings indicate potential differences in the mechanisms underlying the ability of UL23 to promote tumor immune evasion and pathogen immune evasion.

To elucidate the intricacies of how UL23-mediated HCMV enhances PD-L1 expression and fosters immune evasion, we investigated the signaling pathways triggered by HCMV infection and UL23. Our analysis revealed that HCMV and UL23 likely increase PD-L1 expression in GC by activating the PI3K-Akt signaling cascade. However, the precise mechanism underlying this activation remains elusive and is a pivotal area for our future exploration. Investigating the expression of PI3K-Akt-related transcription factors and proteins (e.g., Nrf2, mTOR, etc.) may offer valuable insights into the underlying mechanism. While immunotherapy targeting UL23 is still in its early stages, considering the key role of the interferon response in antiviral immunity and the immunosuppressive microenvironment in GC, UL23 presents potential as a novel target for both antiviral and antitumor therapies.

In summary, we revealed a novel mechanism of HCMV-mediated immune evasion in GC. Specifically, HCMV inhibits CD8^+^ T-cell function in the tumor microenvironment via the PD-1/PD-L1 axis, thereby promoting cancer progression. Our study, however, has limitations. First, we encountered difficulties in obtaining a substantial number of HCMV-positive GC tissue samples, hindering our ability to investigate further the clinical implications of HCMV in immune evasion and immunotherapy. Second, we were unable to generate a recombinant HCMV strain in which UL23 was knocked out, preventing us from exploring the impact of UL23 on PD-L1 expression from a pathogenic perspective. Despite these limitations, our study offers pivotal insights into the role of HCMV in GC development and its underlying mechanisms, paving the way for novel clinical treatment strategies, particularly in the context of immunotherapy for GC.

## Data Availability

No datasets were generated or analysed during the current study.
